# Anoxic spreading depolarization in the neonatal rat cortex *in vitro*

**DOI:** 10.3389/fncel.2023.1106268

**Published:** 2023-03-09

**Authors:** Azat Gainutdinov, Elvira Juzekaeva, Marat Mukhtarov, Roustem Khazipov

**Affiliations:** ^1^Laboratory of Neurobiology, Institute of Fundamental Medicine and Biology, Kazan Federal University, Kazan, Russia; ^2^INMED—INSERM, Aix-Marseille University, Marseille, France

**Keywords:** patch-clamp, membrane potential, ischemia, somatosensory cortex, spreading depolarization, neonate, development

## Abstract

Anoxic spreading depolarization (aSD) is a hallmark of ischemic injury in the cerebral cortex. In adults, aSD is associated with rapid and nearly complete neuronal depolarization and loss of neuronal functions. While ischemia also evokes aSD in the immature cortex, developmental aspects of neuronal behavior during aSD remain largely unknown. Here, using oxygen-glucose deprivation (OGD) ischemia model in slices of the postnatal rat somatosensory cortex, we found that immature neurons displayed much more complex behaviors: they initially moderately depolarized during aSD, then transiently repolarised (for up to tens of minutes), and only then passed to terminal depolarization. The ability to fire action potentials was maintained in neurons mildly depolarized during aSD without reaching the level of depolarization block, and these functions were regained in the majority of immature neurons during post-aSD transient repolarization. The amplitude of depolarization and the probability of depolarization block during aSD increased, whereas transient post-SD repolarization levels and duration, and associated recovery in neuronal firing decreased with age. By the end of the first postnatal month, aSD acquired an adult-like phenotype, where depolarization during aSD merged with terminal depolarization and the phase of transient recovery was lost. Thus, changes in neuronal function during aSD undergo remarkable developmental changes that may contribute to lower susceptibility of the immature neurons to ischemia.

## Introduction

Anoxic Spreading Depolarization (aSD) is a hallmark of ischemic brain injury (Andrew et al., 2022). aSD develops within several minutes after metabolic deprivation and manifests as a wave of collective and nearly complete neuronal depolarization, and depression of electrical activity largely due to depolarization block of action potentials (APs; Somjen, 2001; Dreier and Reiffurth, 2015). aSD is associated with the large negative shift of the extracellular field potential and an increase in tissue light transmittance caused by cytotoxic cell edema (Rader and Lanthorn, 1989; Aitken et al., 1998; Dzhala et al., 2000; Joshi and Andrew, 2001; Juzekaeva et al., 2017, 2018, 2020; Toyoda et al., 2021; Vinokurova et al., 2022). aSD is a highly energy consuming event which aggravates metabolic deficits and initiates intracellular cascades leading to cell death (Somjen, 2001; Strong et al., 2002; Hartings et al., 2003, 2017; Dreier, 2011; Dreier et al., 2013, 2022; Dreier and Reiffurth, 2015). In the OGD model of brain ischemia using submerged brain slices, which simulates the condition of severe global brain ischemia, cortical neurons typically undergo terminal SD to zero mV without recovery (Rader and Lanthorn, 1989; Tanaka et al., 1997; Toyoda et al., 2021; Andrew et al., 2022).

While an immature brain is more tolerant to metabolic deprivation, ischemic/hypoxic brain damage remains a major problem in peri- and neonatology (Saugstad, 2011; Douglas-Escobar and Weiss, 2015). As in adults, aSD occurs during metabolic deprivation in the neonatal cortex and heralds neuronal death. However, in neonates, aSD occurs at much longer delays (up to tens of minutes in the neonatal neocortex and ~1 h in the fetal hippocampus at term) from the onset of hypoxia/OGD than in the adults consistent with higher tolerance of the immature brain to metabolic deprivation (Cherubini et al., 1989; Luhmann and Kral, 1997; Dzhala et al., 2000, 2001; Tyzio et al., 2006), and with lower proneness of the neonatal cortex to SD in general (Bures, 1957; Schade, 1959; Richter et al., 1998; Hertelendy et al., 2019; Andrew et al., 2022). However, aSD in the neocortex during the neonatal period [P0–10 in rodents, which corresponds to a period from mid-gestation to term in human fetus (Khazipov and Luhmann, 2006; Clancy et al., 2007; Colonnese et al., 2010; Colonnese and Khazipov, 2012; Luhmann and Khazipov, 2018; Khazipov and Milh, 2018)] at the cellular level remains largely unexplored. Here, we addressed this question using whole-cell recordings from L4 neurons in slices of the somatosensory whisker-related barrel cortex from 3 to 31 days old rats in the OGD model. We chose this area because it is highly sensitive to ischemia and prone to SD (Lin et al., 1990; Bogdanov et al., 2016; Kaufmann et al., 2017; Juzekaeva et al., 2017, 2020). Our main finding is that neonatal neurons only moderately depolarize during aSD, then transiently repolarise, and only then pass to terminal depolarization. The ability to fire action potentials persists in neurons showing mild depolarization during aSD without reaching the level of depolarization block, and these functions are regained in the majority of immature neurons during post-aSD transient repolarization phase. Thus, immature neurons display protracted in time and complex aSD phenotype with a phase of transient recovery. We suggest that these developmentally unique features of aSD contribute to higher tolerance of the immature brain to ischemia.

## Materials and methods

### Brain slices

Wistar rats (3–31 days old) of either sex were used. Animals were decapitated under isoflurane anesthesia (5%), the brain was rapidly removed and placed in ice-cold (2–5°C) oxygenated (95% O_2_–5% CO_2_) artificial cerebrospinal fluid (ACSF) of the following composition (in mM): NaCl 126, KCl 3.5, CaCl_2_ 2, MgCl_2_ 1.3, NaHCO_3_ 25, NaH_2_PO_4_ 1.2, and glucose 20 (pH 7.4). Four hundred μm thick thalamocortical slices were cut using a PELCO easiSlicer^TM^ vibratome (Ted Pella, Inc., Redding, CA, USA). A total of *n* = 58 slices obtained from 43 rats were used (1 slice/rat: *n* = 29 rats; 2 slices/rat: *n* = 13 rats; 3 slices/rat: *n* = 1 rat). Data from all slices were pooled for analysis; n indicates the number of slices. Slices containing the barrel cortex were selected by anatomical coordinates (Khazipov et al., 2015) and the presence of barrel structures in L4. Slices were first kept in ACSF for 30 min at 32°C and then at room temperature (20–22°C) for at least 1 h before use. For recordings, slices were placed into a submerged chamber and superfused with oxygenated ACSF at 30–32°C at a flow rate of 10 ml/min. Oxygen/glucose deprivation (OGD) was induced by superfusion with ACSF in which N_2_ replaced O_2_ and sucrose replaced glucose at equimolar concentration.

### Electrophysiological recordings

For electrophysiological recordings, slices were placed in the recording chamber under an upright microscope BX51WI (Olympus, Tokyo, Japan) equipped with a dry 4×/0.10 Plan N objective and a water immersed 40×/0.80 LUMPlanFL N objective. L4 neurons were identified at 40× magnification using infrared-differential interference contrast (IR-DIC) microscopy. Visual patch-clamp recordings were performed using a MultiClamp 700B (Axon Instruments, Union City, CA, USA) amplifier as described previously (Juzekaeva et al., 2018). Patch electrodes were made from borosilicate glass capillaries (BF150-86-10, Sutter Instrument, Novato, CA, USA) and had a resistance of 4–7 MΩ. The pipette (intracellular) solution contained (in mM) 131 potassium gluconate, 4 KCl, 10 HEPES, 10 phosphocreatine, 4 MgATP, and 0.3 Na_2_GTP (adjusted to pH 7.3 with KOH). 200-ms suprathreshold depolarizing current pulses were applied every 10 s to examine the ability of neurons to fire APs and monitor the membrane resistance (*R_m_*). Extracellular DC recordings of the local field potentials (LFP) were performed in the barrel cortex using glass pipette electrodes pulled from borosilicate glass capillaries (BF150-86-10, Sutter Instrument, Novato, CA, USA) with resistances of 2–3 MΩ when filled with ACSF. LFP recordings were performed in voltage-clamp DC mode, then currents were inverted and voltage calibrated using 5 mV steps. Extracellular and patch-clamp recordings were digitized at 32 kHz with a Digidata 1440A interface card (Axon Instruments) and analyzed offline using MATLAB (MathWorks, Natick, MA, USA) routines. Optical intrinsic signal (OIS) recordings were performed at 4× magnification. The boundaries of layer 4 were discerned by barrel-shaped structures at 4× magnification and high cell density in layer 4 at 40× magnification. The slice was illuminated by a halogen lamp with a 775 nm bandpass filter. Images were acquired using a QIClick-R-F-M-12 CCD camera (QImaging, Surrey, BC, Canada) at 696 × 520 or 1,392 × 1,040 pixel resolution at 1 frame/2.5 s acquisition rate.

### Data analysis

Experimental data were processed using MATLAB environment (MathWorks, USA). Matlab’s Signal Processing Toolbox functions were used for peak detection procedures. Patch-clamp *E_m_* recordings were down-sampled to 1 Hz and filtered using a median filter within sliding 30 s window with 1 s time step to eliminate responses to depolarizing current steps. aSD was detected as a wave of *E_m_* depolarization occurring in association with characteristic negative LFP shifts and OIS signals (Juzekaeva et al., 2020). The time corresponding to the peak depolarization during aSD was defined as aSD time. aSD amplitude was defined as a difference between *E_m_* at the aSD peak and the baseline *E_m_* values between -3 and -1 min time interval before the aSD peak. The term of transient recovery (TR) was applied for a phase of transient *E_m_* repolarization between aSD and terminal depolarization (TD). TR duration was defined as a time period between aSD peak (TR onset = time of aSD peak) and transition to TD was determined at the peak of the first *E_m_* derivative (d*E_m_*/dt, dt = 1 min). The peak of *E_m_* repolarization during TR was taken as the TR time reference point. Action potentials (AP) in response to suprathreshold current steps were detected from raw signals using *peak detection* function as events with the peak prominence of >3 mV and 5 ms minimum inter-peak interval. Two neurons at P25 with the electrophysiological phenotype of fast spiking interneurons showed no differences in response to OGD and were pooled with other neurons in the P25–31 age group. DC LFP signals were down-sampled to 1 kHz and low-pass filtered using 1 s sliding window. OIS was calculated using the first-frame subtraction approach: OIS (t) = (I (t) − I0)/I0, where I (t)—pixel intensity at the moment t, I0—time-averaged pixel intensity in the preconditioned control period (1 min). The resulting frames were filtered with a 2 × 2 Gaussian filter. Regions of interest (ROIs) were selected as square areas near the recording site. OIS traces were calculated as the average OIS signal in the selected ROIs.

### Statistical analysis

Statistical analysis was performed using the MATLAB Statistics toolbox. Pooled data are presented as median, 25th (Q1), and 75th (Q3) percentiles in boxplots or shaded areas. A two-sided Wilcoxon rank sum test was performed to assess the difference between groups. For testing binomial data and proportions we used Two Sample Z-Test for Proportions. Correlations were calculated as Spearman’s correlation coefficient with the exact *p*-value. The level of significance was kept at *p* < 0.05.

## Results

**Figure 1:**
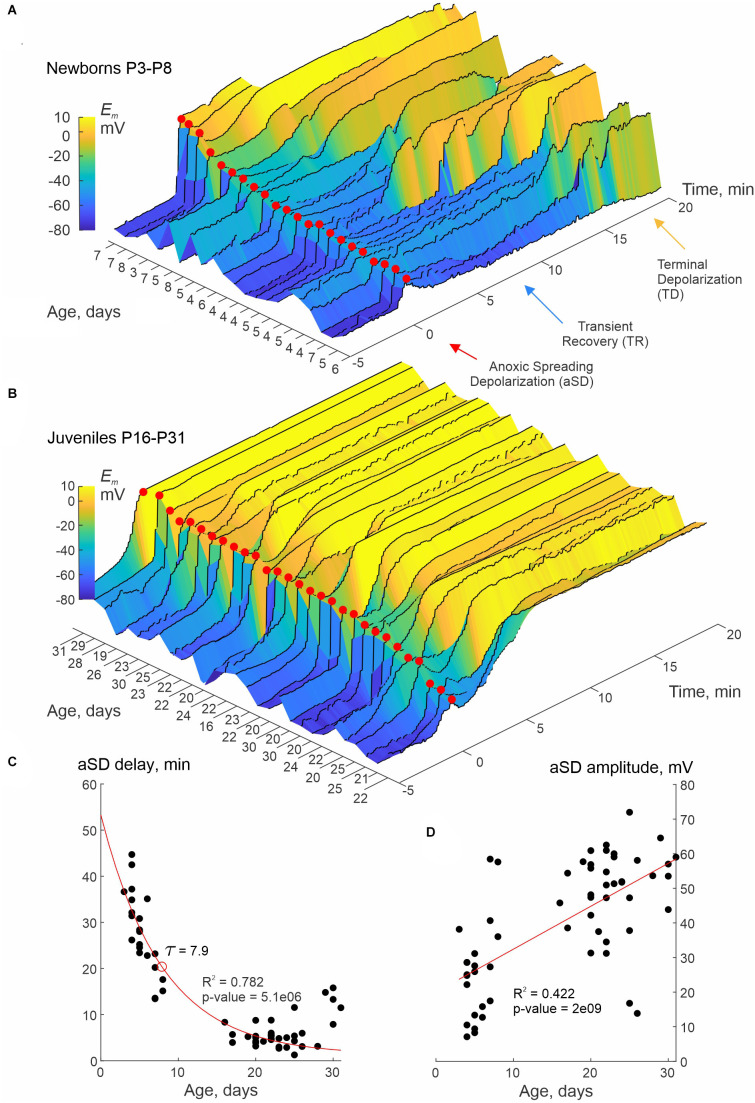
Smaller aSD amplitude and transient recovery of membrane potential in neurons of newborn rat cerebral cortex. **(A,B)** Dynamics of membrane potential (*E_m_*) in L4 neurons recorded in whole-cell current-clamp mode during oxygen-glucose deprivation (OGD) in slices of somatosensory cortex prepared from neonatal (P3–8) **(A)** and juvenile (P16–31) **(B)** rats. The *E_m_* signal was low-pass filtered with a median filter using a sliding window of 30 s and increment of 1 s to eliminate spikes and the responses to depolarizing current steps. Color-coded *E_m_* values are plotted with reference to aSD (*T* = 0, red dots). Each trace corresponds to an individual neuron, the age of the animal is indicated on the left horizontal axis. Note the smaller aSD amplitude and the phase of transient *E_m_* recovery (TR) before the transition to terminal depolarization (TD) in the neonatal group. Pooled data from *n* = 22 **(A)** and *n* = 29 **(B)** neurons. In some cases, the recordings stopped when the TD reached a plateau. **(C,D)** Delay of aSD peak from the OGD onset **(A)** and aSD amplitude **(B)** as a function of age. Each dot corresponds to an individual neuron. The aSD amplitude was calculated from the baseline *E_m_* values between −3 and −1 min time interval before the aSD peak. The red lines show exponential **(A)** and linear **(B)** regression. Pooled data from *n* = 58 neurons from P3 to P31 rats.

In the present study, we explored the dynamics of the resting membrane potential (*E_m_*) and neuronal firing during OGD in slices of the barrel cortex of rats aged from P3 to P31 using whole-cell current-clamp recordings of L4 neurons and concomitant extracellular recordings of LFP, and optical intrinsic signals (OIS) imaging. Perfusion with OGD-solution evoked aSD in all neurons at all ages. However, aSD amplitude and aSD delay after OGD onset, and post-aSD *E_m_* dynamics were variable and age-dependent (Figure 1). *E_m_* dynamics aligned against aSD peak (marked by red dots) and sorted by the aSD amplitude in neurons from newborn (P3–8) and juvenile (P16–31) rats are shown in Figures 1A,B, respectively. Overall, in the newborn animals, aSD was smaller in amplitude, and was typically followed by a remarkable phase of transient recovery (TR) of *E_m_* before a passage to terminal plateau depolarization (TD) near 0 mV. The level and duration of TR were more pronounced in the cases with low-amplitude aSDs, where TR could last for up to 15 min. Only a few neurons with large-amplitude aSD showed “adult-like” aSD phenotype of permanent depolarization without TR in the newborn animals. In contrast, neurons in juvenile rats typically displayed large amplitude aSDs without TR, or, less frequently, with a short and small TR (Figure 1B). At the group level, aSD delay from the OGD onset shortened (Figure 1C), whereas aSD amplitude increased (Figure 1D) with the animals’ age. Thus, aSD in the immature neurons is smaller in amplitude and displays a unique intermediate phase of TR before a passage to TD.

**Figure 2:**
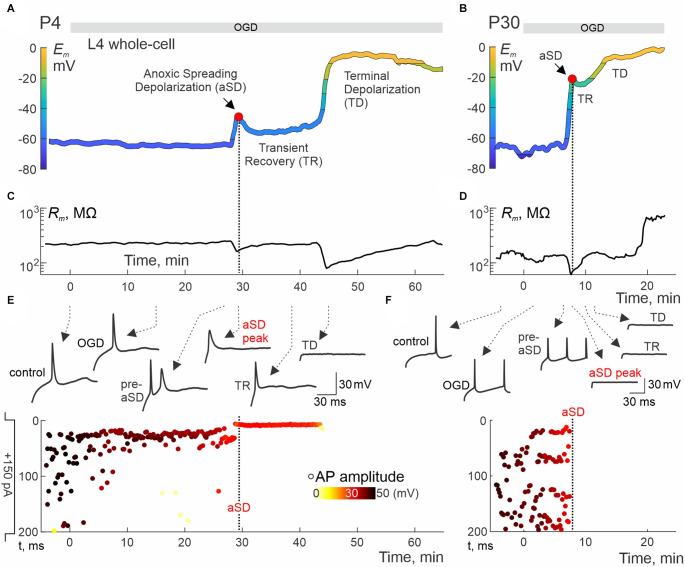
Immature neurons remain excitable during aSD and transient recovery phase. **(A,B)** Examples of *E_m_* recordings from L4 neurons in slices from P4 **(A)** and P30 **(B)** rats. *E_m_* traces are low-pass filtered and color-coded as in Figure 1. The exposure to OGD is indicated by the horizontal gray bar at the top. **(C,D)** Corresponding changes in input resistance (*R_m_*). **(E,F)** Corresponding example responses to suprathreshold current steps at different time points indicated by arrows (top traces) and raster plots of APs evoked by suprathreshold (+150 pA, 200 ms) current steps (y-axis shows depolarizing step time (top: step start; bottom: step end); APs amplitude is color-coded) (bottom plots). Full trace examples are shown in Supplementary Figure 1. Note that the AP firing is maintained through the aSD and TR, and disappears only during the transition to TD in the P4 neuron, whereas in the P30 neuron APs irreversibly disappear as soon as aSD occurs. *T* = 0 on the x-scale corresponds to the onset of OGD.

**Figure 3:**
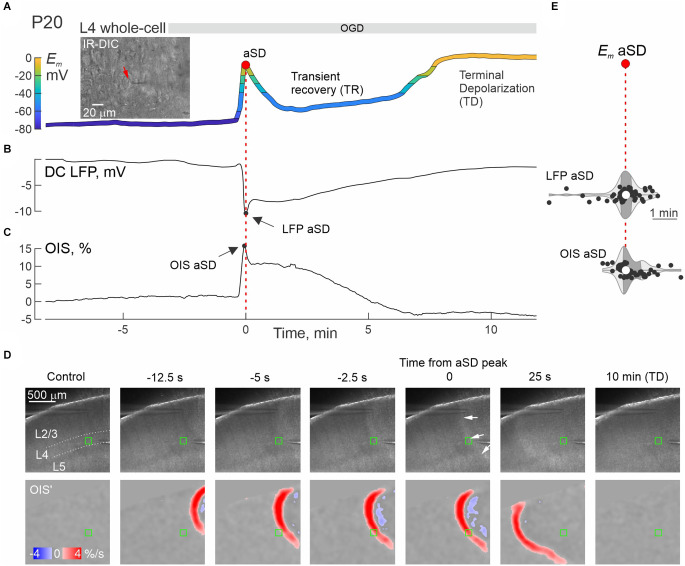
aSD correlates with negative LFP shifts and an increase in optical tissue transparency in the developing cortex. **(A–C)** Simultaneous whole-cell *E_m_* recordings from an L4 neuron **(A)**, extracellular DC-LFP recordings **(B)**, and OIS recordings **(C)** near the recorded neuron during OGD in a P20 rat somatosensory cortex slice. *T* = 0 indicates aSD peak during whole-cell recordings (*E_m_*—aSD). The inset on panel **(A)** shows an IR-DIC image of layer 4 (40x magnification), the neuron being recorded is marked with a red arrow. **(D)** Microphotograph of a slice (4x magnification, raw data, top panels) and the OIS first derivative (bottom panels) at different delays from aSD recorded from the L4 neuron shown on panel **(A)**. The region of interest (ROI) for OIS quantified in panel **(C)** is indicated by a green rectangle. **(E)** Distribution of aSD delays recorded by extracellular electrodes (LFP—aSD) and OIS (OIS—aSD) from aSD during whole-cell recordings (*E_m_*—aSD, vertical dotted line). Hereafter, each dot corresponds to an individual cell/slice, the violin plots show the probability density of the data at different values smoothed by the kernel density estimator, the white circles are the median, and the dark shaded area is the interquartile range. Pooled data from *n* = 58 cells/slices from P3 to P31 rats. There was no significant time lag between LFP—aSD (*p* = 0.54), OIS—aSD (*p* = 0.93, Wilcoxon rank sum test), and *E_m_*—aSD.

We further explored changes in neuronal firing by injecting suprathreshold depolarizing current steps. Exemplary whole-cell recordings from L4 neurons in P4 and P30 rats are presented in Figure 2. P4 neuron displayed small-amplitude aSD associated with a mild transient drop in membrane resistance (*R_m_*) followed by 10 min-long TR with a regain in *R_m_* values (Figures 2A,C). TR was followed by nearly complete depolarization during TD. Action potentials (APs) could be reliably evoked by depolarizing current in this neuron not only through the pre-aSD period, but also during aSD and TR phase (*nota bene*: no depolarization block in this case!), and were lost only after a passage to TD (Figure 2E, Supplementary Figure 1). In a P30 neuron (Figure 2B), membrane potential rapidly dropped to −20 mV, then showed brief and little recovery during TR phase, followed by TD to 0 mV, during which *R_m_* displayed an apparent increase to ~1 GOhm (Figure 2D) presumably reflecting a passage from whole-cell to outside-out due to tissue displacement caused by aSD-triggered edema (Juzekaeva et al., 2018, 2020). In the P30 neuron, APs were completely suppressed, without any recovery starting from aSD (Figure 2F, Supplementary Figure 1). Thus, while the P30 neuron displayed an “adult-like” phenotype of rapid and irreversible *E_m_* loss during aSD together with an irreversible depression of APs, P4 neuron showed persistence of APs during aSD and TR, pointing to a qualitative difference of aSD in the immature neuron. All aSDs of different amplitudes were associated with a characteristic negative local field potential shift (Figure 3B), and a concomitant wave of increase in the brain tissue light transparency propagating across the entire slice of the cortex during OIS recordings (Figures 3C,D). Peak depolarization during aSD in L4 neurons was attained simultaneously with the peak negativity of the local field potential and the peak of light transparency during aSD wave in the vicinity of the recorded neuron (Figure 3E).

**Figure 4:**
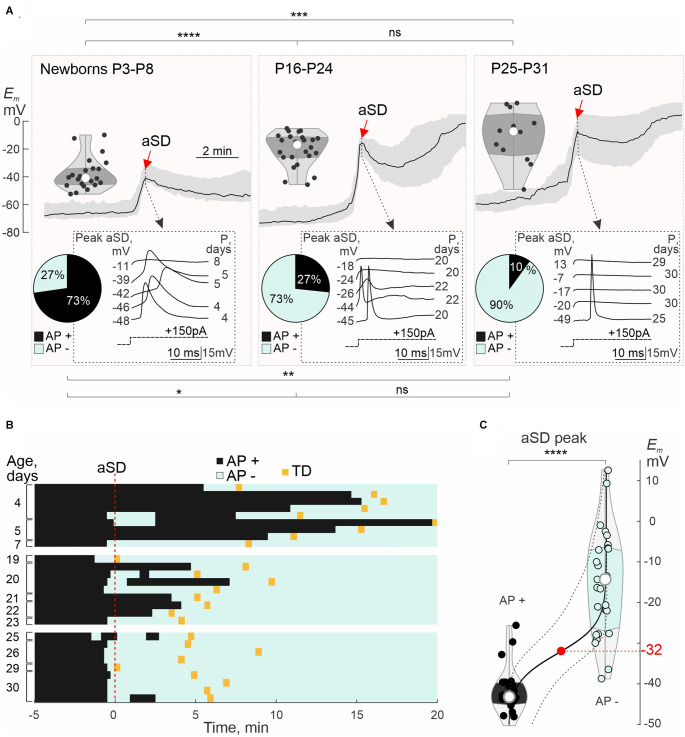
Postnatal changes in aSD peak value and neuronal firing during aSD: group data. **(A)** Top traces, grand average aSD (black trace—median *Em*; shaded area—interquartile range) and corresponding statistical plots of *E_m_* values at the aSD peak in L4 neurons from newborn (P3–P8, *n* = 22 cells), and juvenile rats (P16–P24, *n* = 24 cells; and P25–P31, *n* = 12 cells). Gray insets below, examples of responses to depolarizing current steps at the aSD peak in five neurons from each age group, with the corresponding *E_m_* aSD peak value (left) and age (right). Circular histograms show neuronal firing at the aSD peak (black—AP+ neurons responding with APs to depolarizing steps, cyan—AP− neurons not showing APs in response to depolarizing steps). Note that in newborn animals, neuronal depolarization reaches only a weak level during aSD, that most newborn neurons retain neuronal firing during aSD, and that ability to fire APs during aSD diminishes with age. **(B)** Representative raster plots of APs evoked by depolarizing current steps (inter-step interval 10 s) in L4 neurons of different age groups during OGD-induced aSD (*T* = 0, vertical red dashed line). The black and cyan bins denote responses with AP (AP+) and without AP (AP−), respectively. The transition to TD is indicated by a yellow rectangle. **(C)** Group data on neuronal firing during aSD as a function of peak *E_m_* depolarization during aSD. The black line is a binomial logistic regression fit, dashed lines are 95% confidence intervals for prediction. Half-probability of AP inactivation (or depolarization block) occurs during aSD attaining *E_m_* = −32 (−39/−24) mV [median (interquartile range)]. *, *P* < 0.05; **, *P* < 0.01; ***, *P* < 0.0001; ****, *P* < 0.00001; ns, not significant (Wilcoxon rank sum test).

We next analyzed in detail the age-dependence in the depolarization level attained during aSD and associated changes in the neuronal firing (Figure 4). In the newborn animals (P3–8), neurons depolarized, on average, to ~−40 mV, and APs persisted in their vast majority (73%; *n* = 16 of 22 cells) at the aSD peak (Figure 4A, see also AP raster plots on Figure 4B). In the juvenile rats, peak depolarization during aSD attained on average −18 mV at P16–24, and −7 mV at P25–31. Along with a developmental increase in the aSD *E_m_* levels, the proportion of neurons maintaining the ability to fire APs during aSD decreased to 27% at P16–24 (*n* = 4 of 15 cells), and to 10% at P25–31 (*n* = 1 of 10 cells; Figures 4A,B). Loss of neuronal firing depended on the depolarization level attained during aSD within all age groups, as illustrated by responses to depolarizing steps in different cells on insets in Figure 4A. To estimate the AP inactivation threshold, we analyzed neuronal firing during the rising aSD phase. We found that failure in evoking APs by depolarizing current steps occurred when *E_m_* attained ~−30 mV (Figure 4C). Thus, loss of neuronal firing during aSD critically depends on whether neuronal depolarization attains AP inactivation threshold, which barely occurs in the newborn neurons showing mainly moderate depolarization during aSD.

**Figure 5:**
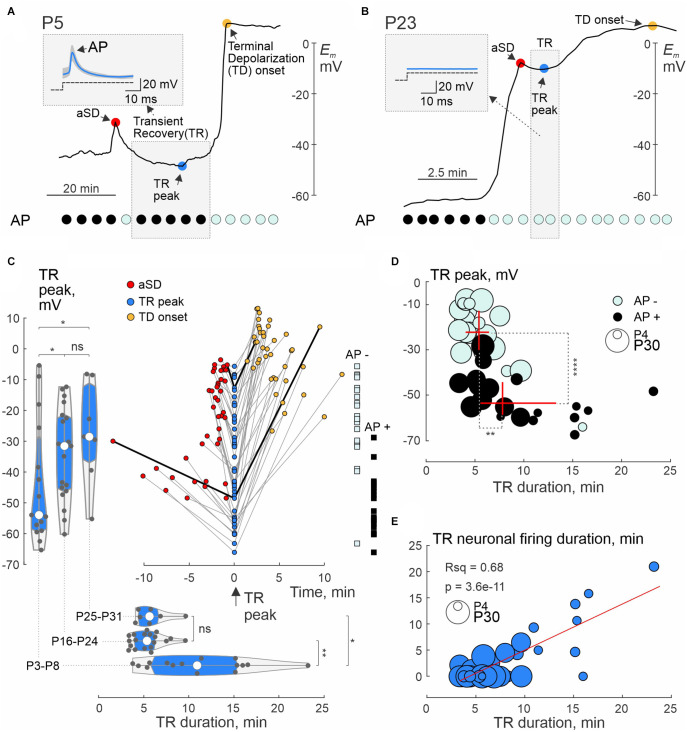
Postnatal changes in transient membrane potential recovery following aSD. **(A,B)** Examples of whole-cell recordings from L4 neurons in P5 **(A)** and P23 **(B)** rats. Red circle—peak aSD; blue circle—peak negativity during TR; TD—terminal depolarization. Top left insets—mean responses to depolarizing current steps during TR (median blue traces are superimposed on all gray traces). At the bottom are corresponding graphs indicating responses with APs (black circles) and without APs (cyan circles). Note the long TR with preservation of APs after a small aSD at P5, and weak short TR without APs at P23. **(C)** Summary plot of *E_m_* values achieved during TR, aSD, and TD in individual cells. The peak of negativity during TR is taken as a time reference point. TR, aSD, and TD from individual neurons are connected by gray lines, and values connected by black lines correspond to the examples shown in panels **(A)** and **(B)**. The left and bottom histograms show the distribution of maximum *E_m_* negativity achieved during TR and TR duration, respectively, in the three age groups. On the right, the distribution of TR—*E_m_* values according to the absence (AP−, cyan squares) and presence (AP+, black squares) of APs during TR. **(D)** Relationship between the TR—*E_m_* level and duration of TR as well as neuronal firing during the TR phase. Black (AP+) and cyan (AP−) circles indicate cells that showed or did not show APs in response to depolarizing steps during the TR phase, respectively. The age of the animal is coded by the size of the circle. The red crosses show the median ± interquartile range of TR—*E_m_* levels and duration of TR in the AP+ and AP− cell groups. Note the presence and longer persistence of neuronal firing in cells with more negative *E_m_* values reached during the TR phase. **(E)** The duration of the period within the TR phase, during which neurons retain the ability to fire APs in response to depolarizing steps, correlates with the duration of TR. The red line shows a linear regression fit. **(C–E)**: Pooled data from P3 to P8 (*n* = 15), P16–P23 (*n* = 19) and P25–P31 (*n* = 7). Seventeen cells without TR were excluded from the analysis. *, *P* < 0.05; **, *P* < 0.01; ****, *P* < 0.0001; ns, not significant (Wilcoxon rank sum test).

The phase of transient recovery in *E_m_* and neuronal firing also showed age-dependence. Neurons from newborn rats displayed more negative TR *E_m_* values and longer duration TR phase, and more frequently maintained AP firing than neurons from the juvenile group (Figures 5A,B; see also Figures 2 and 4B). Group data on the amplitude and time of TR at its peak negativity (the latter taken as a time reference point) and corresponding values of aSD and TD at their onset, neuronal firing during the TR phase, and age-dependence of TR amplitude and duration are summarized in Figure 5C. Noteworthy, the level and duration of TR negatively correlated with aSD amplitude and were more prominent in the newborn animals (Figure 5C, left and bottom violin plots). Also, TR lasted longer in cells with more negative levels of *E_m_* recovery, and this was associated with longer duration of neuronal firing during the TR phase (Figures 5D,E). Similar to aSD, the ability of cells to fire APs during the TR phase depended on whether the cell repolarized below the AP inactivation threshold at around -30 mV, which typically occurred in the newborn group (Figures 5C,D). The final TD *E_m_* values also showed an age dependence, reaching more negative values at P3–P8: −7.6 (−14.7 − +0.4) mV (*n* = 17) than at P16–P24: +4.82 (−2.40 − +7.6) mV (*n* = 22; *p* = 0.00055) and at P25–P31: +10.5 (+0.0 − +14.6) mV (*n* = 11; *p* = 0.00038).

## Discussion

The main finding of the present study is that aSD has several developmentally unique features, including graded levels of depolarization and long-lasting post-aSD phase of transient repolarization preceding TD in the newborn cortical neurons. Furthermore, neuronal functions such as the ability to generate APs are barely depressed during neonatal aSD and TR phase. These results highlight a critical developmental difference in the response of the immature brain to metabolic deprivation and significantly broaden the SD spectrum from a developmental perspective.

Based on studies in the adult brain, aSD has been classically viewed as a binary process, during which neurons rapidly and completely lose their membrane potential (Rader and Lanthorn, 1989; Tanaka et al., 1997; Somjen, 2001; Dreier and Reiffurth, 2015; Toyoda et al., 2021; Andrew et al., 2022). Generative mechanisms of aSD are fundamentally similar to SD in other models and conditions, and involve an increase in extracellular potassium, glutamate, and activation of voltage and ligand—gated conductances (Leao, 1944, 1947; Somjen, 2001; Dreier, 2011; Pietrobon and Moskowitz, 2014; Ayata and Lauritzen, 2015; Dreier and Reiffurth, 2015; Hartings et al., 2017; Andrew et al., 2022). While *E_m_* repolarizes after SD in the metabolically preserved tissue with a pivotal contribution of Na, K—ATPase, under conditions of metabolic deprivation aSD is associated with permanent depolarization as energy sources to fuel Na, K—ATPase are limited. We found that in the immature neurons, aSD was associated with only mild levels of depolarization and was followed by transient repolarization during TR-phase. The stationary state of complete *E_m_* loss, which characterizes full-size aSD in adult neurons, was achieved only during the subsequent TD-phase. Small amplitude aSD likely produces smaller disturbance in the ionic gradients and thus less aggravates the metabolic status of the immature neurons than full-size aSD, leaving energy sources fueling Na, K—ATPase to restore *E_m_* after aSD during the TR phase. This is consistent with the inverse relationships between aSD amplitude and TR: indeed, TR was more prominent in neurons with small amplitude aSDs. Several developmental factors may contribute to unique aSD features in neonates. First, immature neurons have lower metabolic rate and thus are more tolerant to metabolic deprivation consistent with much longer aSD delays than in adults (Cherubini et al., 1989; Luhmann and Kral, 1997; Dzhala et al., 2000, 2001; Tyzio et al., 2006). Second, cortex in the newborns is less prone to SD in general, that could be due to large extracellular space, low levels of synaptic connectivity, the small density of ionic channels, and their different developmental properties (Bures, 1957; Schade, 1959; Richter et al., 1998; Hertelendy et al., 2019; Andrew et al., 2022). We propose that a combination of these developmental factors endows immature neurons with on one hand, higher tolerance to metabolic deprivation, and on the other hand—a lower ability to support mutual depolarization *via* synaptic and ephaptic mechanisms in the SD genesis, explaining small aSD amplitude and TR in the immature neurons. Importantly, these unique developmental aSD features could be only revealed in the present study using whole-cell *E_m_* recordings. aSD *per se* was also reliably detected as a characteristic negative shift of the extracellular field potential and increase in tissue transparency during OIS recordings [Figure 3 and References Luhmann and Kral, 1997; Dzhala et al., 2000, 2001; Tyzio et al., 2006)], but the magnitude of these signals weakly correlated with the level of neuronal depolarization during aSD (Supplementary Figure 1). Also, TR and TD did not have clear manifestations during extracellular field potential and OIS recordings. Because we performed whole-cell recordings from only one neuron in the preparation, we cannot conclude whether the level of depolarization during aSD, level and duration of TR, and transition to TD were uniform in the entire neuronal population. Moreover, the lack of extracellular LFP and OIS manifestations suggests that TR duration and transition to TD are likely desynchronized between neurons. The desynchronized transition to TD in the immature cortex may result from metabolic heterogeneity between neurons (e.g., due to their different age) and hence the different abilities to maintain *E_m_* during TR, greater extracellular space, low levels of synaptic connectivity, and low density of ion channels and hence smoother increases in extracellular glutamate and potassium. In future studies, it would be of interest to test this hypothesis using multiple whole-cell recordings, or calcium/voltage sensitive dye imaging. The latter approach could be advantageous in overcoming a problem of tissue swelling and displacement that starts after aSD and may cause loss of contact of the electrode with cell and apparent loss of *E_m_* during whole-cell recordings including a transition from whole-cell to outside-out configuration (Figure 2B and Juzekaeva et al., 2020). This artifact can lead to an underestimation of the TR duration, which may in fact be even longer than reported here, and can also affect the measurement of TD *E_m_* values. Interestingly, in the P25-P31 age group, we observed that TD *E_m_* reached positive values of ~+10 mV, which may be due to a greater concentration of impermeable anions in the extracellular than in the intracellular space, a consequence of swelling and shrinkage of the extracellular space.

Our study also revealed that aSD in the neonates lacks the hallmark SD feature—depression of APs. In adults, at the aSD onset, when *E_m_* approaches values close to the AP threshold cortical neurons typically generate one or few APs, and then permanently lose neuronal firing upon further depolarization as a result of voltage-gated sodium channels inactivation and AP depolarization block (Grafstein, 1956; Dzhala et al., 2000, 2001; Canals et al., 2005). Synaptic activity may also display “paradoxical” recovery from non-spreading depression at the aSD onset followed by complete suppression of synaptic responses (Sick et al., 1987; Fowler, 1992; Khazipov et al., 1993; Zhu and Krnjevic, 1999; Dzhala et al., 2001). In contrast, we found that ability to fire APs persists during aSD, and that APs can be reliably triggered during post-aSD TR phase in the majority of neonatal neurons. Our results indicate that the persistence of neuronal firing is primarily due to mild levels of depolarization which barely reaches the threshold of AP inactivation in the immature neurons. Altogether, these findings point to significant developmental differences in aSD properties in immature neurons. We also propose that neonatal aSDs described in the present study broaden the existing SD spectrum (Somjen, 2001; Pietrobon and Moskowitz, 2014; Ayata and Lauritzen, 2015; Dreier and Reiffurth, 2015; Hartings et al., 2017) by adding to a family of full-sized SDs an immature phenotype with its notable mild depolarization levels, TR phase and lack of loss in neuronal functions. In future research, it would be interesting to determine the mechanisms underlying this peculiar phenotype of immature aSD, including the computational modeling approach (Zandt et al., 2013; Herreras and Makarova, 2020; Kalia et al., 2021).

How these unusual aSD features may impact hypoxic/ischemic injury in the neonatal brain? There is general agreement that aSD is a critical event during metabolic deprivation (Andrew et al., 2022). In adult brain, aSDs are highly energy consuming events associated with a profound depletion of energy sources under conditions of a limited metabolic supply (Somjen, 2001; Strong et al., 2002; Hartings et al., 2003, 2017; Dreier et al., 2013, 2022; Dreier and Reiffurth, 2015). Mild level aSDs in the neonatal neurons likely less disturb ionic gradients and thus consume less energy for their restoration. Therefore, it is conceivable that the inability to generate full-size aSDs is beneficial to the neonatal brain and may be a factor contributing to higher tolerance of the neonatal brain to hypoxia/ischemia. Indeed, a similar correlation between weak depolarization and higher tolerance to OGD was also observed in the adult hypothalamus (Brisson and Andrew, 2012). Yet, factors increasing energy consumption such as adenosine A1 receptor antagonists and drugs provoking epileptiform activity are known to accelerate the occurrence of aSD and neuronal death whereas energy-savers such as NKCC1 antagonists and oxytocin exert beneficial actions in the neonatal brain during metabolic deprivation (Dzhala et al., 2000; Tyzio et al., 2006; Khazipov et al., 2008). Even less energy consuming, immature aSD still should jeopardize metabolic status, and inevitably leads to delayed terminal depolarization if metabolic supply is not restored. Indeed, the transition to TD occurred faster in neurons with larger-size aSD. This suggests that aSD occurring simultaneously in the entire population of neurons recruited by aSD wave opens a time window during which neurons attain their cell-specific “commitment point” of irreversible *E_m_* loss and neuronal death at variable delays determined by the aSD size that is consistent with variable survival of neurons following aSD in the juvenile cortex (Juzekaeva et al., 2020). In future studies, it would also be interesting to determine how developmental changes in the composition of the intracellular milieu (notably chloride concentration) and extracellular metabolites (ketone bodies) influence the developmental changes in the aSD described in this study.

In conclusion, our study provides descriptions of aSD in the neonatal cortex at the cellular level. We have shown that aSD in the newborn cortical neurons displays several unique features, including mild levels of depolarization and transient depolarization. We also found that neuronal functions, including the ability to generate APs persist during aSD and TR in neurons with mild aSD. These results highlight a critical developmental difference in the response of the immature brain to metabolic deprivation and are important for understanding the development of cortical damage during hypoxia/ischemia in the neonatal brain.

## Data availability statement

The raw data supporting the conclusions of this article will be made available by the authors, without undue reservation.

## Ethics statement

The animal study was reviewed and approved by Local Ethical Committee of Kazan Federal University (#24/22.09.2020) and French National Institute of Health and Medical Research (APAFIS #16992-2020070612319346 v2). The animal experiments were carried out in compliance with the ARRIVE guidelines. Animal care and procedures were in accordance with EU Directive 2010/63/EU for animal experiments.

## Author contributions

RK conceptualized and drafted the manuscript. EJ, AG, and MM performed the experiments. AG analyzed data. AG, EJ, MM, and RK revised the manuscript and interpreted the data. All authors contributed to the article and approved the submitted version.
