# Supplementary figures and images for: Anoxic spreading depolarization in the neonatal rat cortex in vitro
Source: Front Cell Neurosci. 2023 Mar 9;17:1106268. doi: 10.3389/fncel.2023.1106268 (PMC10034194; doi:10.3389/fncel.2023.1106268)

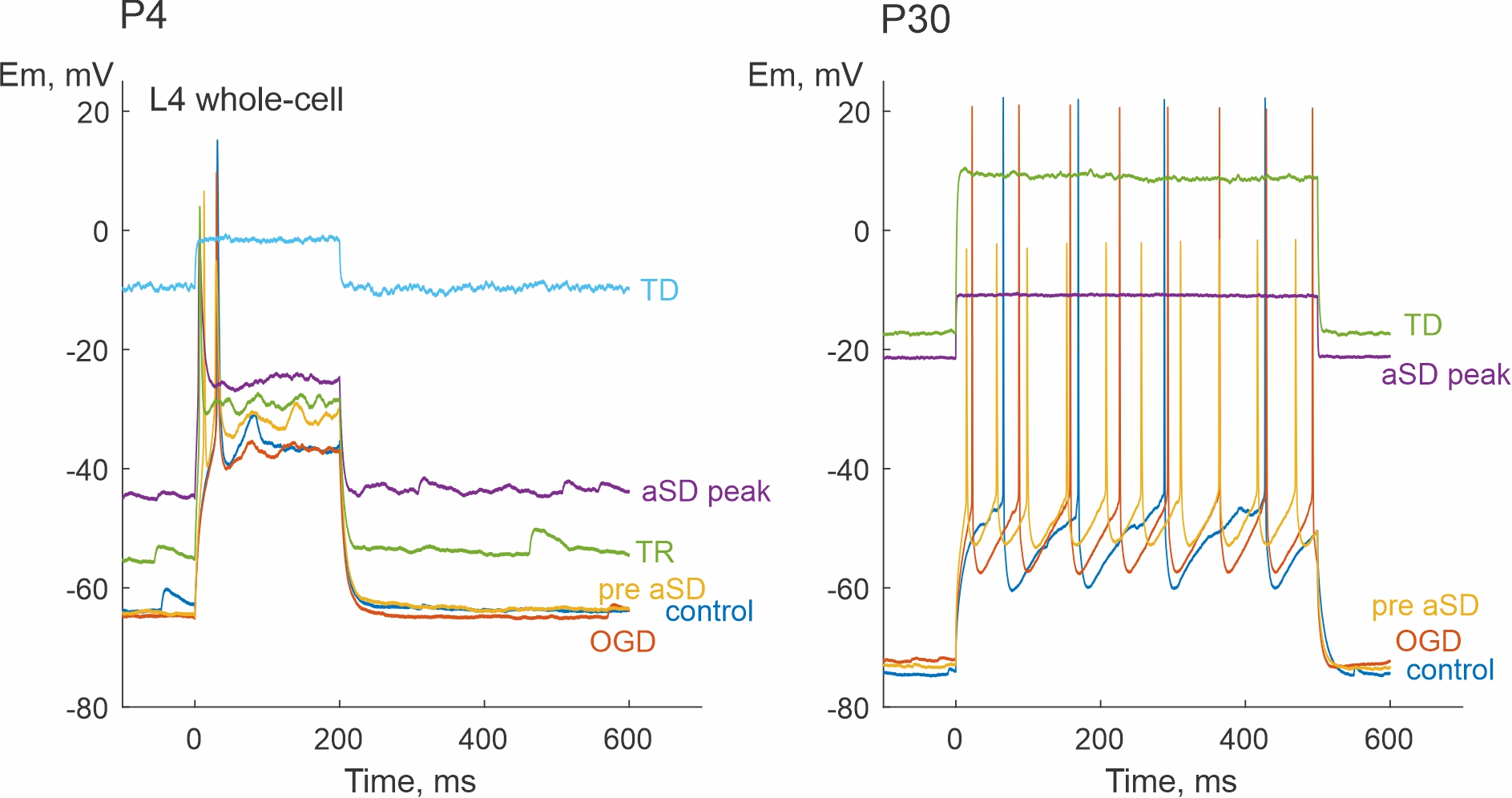

Supplement: Supplementary file 1 [file Image_1.JPEG]

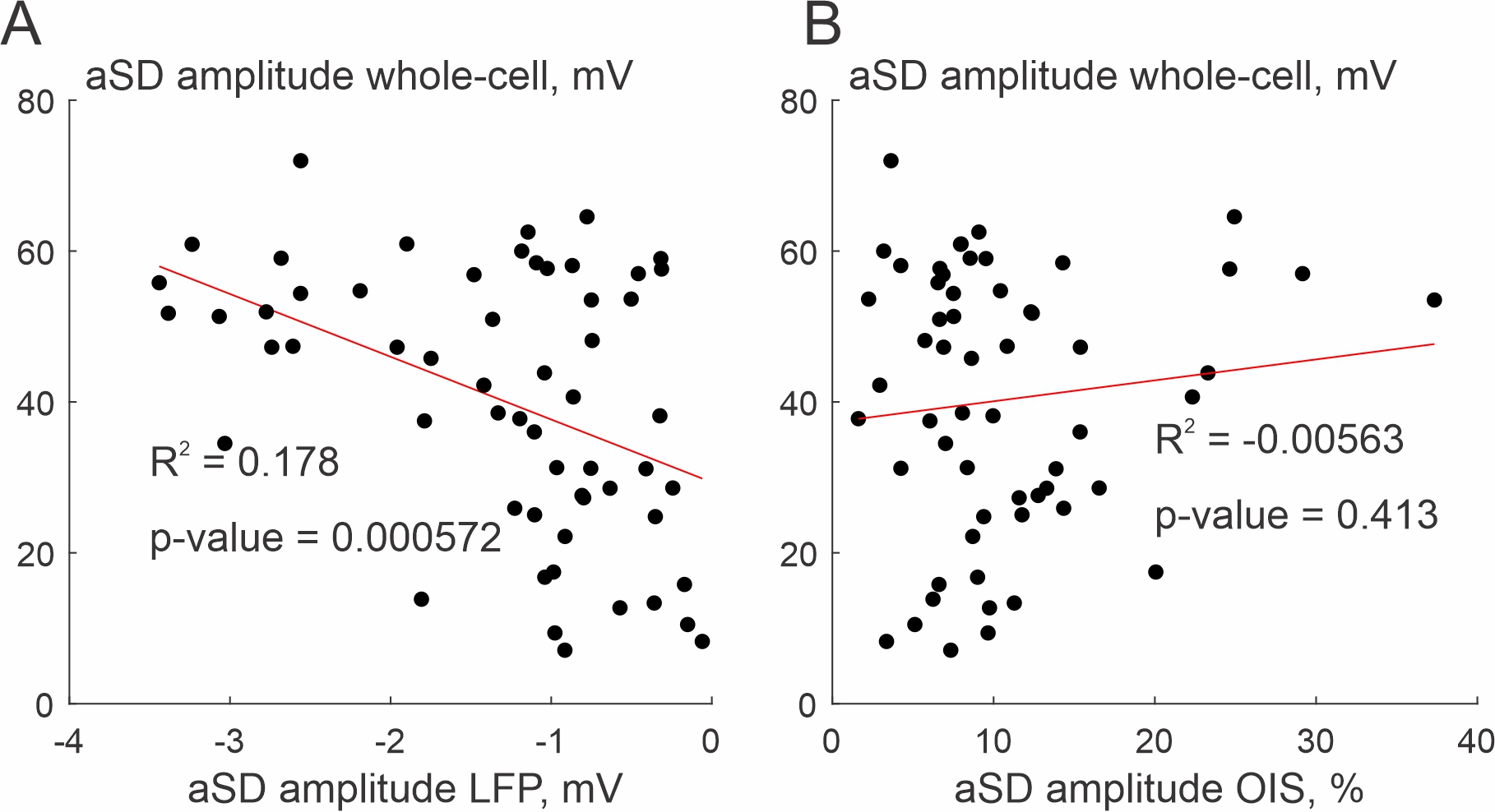

Supplement: Supplementary file 2 [file Image_2.JPEG]
